# Evidence on the Formation of Singlet Oxygen in the Donor Side Photoinhibition of Photosystem II: EPR Spin-Trapping Study

**DOI:** 10.1371/journal.pone.0045883

**Published:** 2012-09-26

**Authors:** Deepak Kumar Yadav, Pavel Pospíšil

**Affiliations:** Department of Biophysics, Centre of the Region Haná for Biotechnological and Agricultural Research, Faculty of Science, Palacký University, Olomouc, Czech Republic; Consejo Superior de Investigaciones Cientificas, Spain

## Abstract

When photosystem II (PSII) is exposed to excess light, singlet oxygen (^1^O_2_) formed by the interaction of molecular oxygen with triplet chlorophyll. Triplet chlorophyll is formed by the charge recombination of triplet radical pair ^3^[P680^•+^Pheo^•−^] in the acceptor-side photoinhibition of PSII. Here, we provide evidence on the formation of ^1^O_2_ in the donor side photoinhibition of PSII. Light-induced ^1^O_2_ production in Tris-treated PSII membranes was studied by electron paramagnetic resonance (EPR) spin-trapping spectroscopy, as monitored by TEMPONE EPR signal. Light-induced formation of carbon-centered radicals (R^•^) was observed by POBN-R adduct EPR signal. Increased oxidation of organic molecules at high pH enhanced the formation of TEMPONE and POBN-R adduct EPR signals in Tris-treated PSII membranes. Interestingly, the scavenging of R^•^ by propyl gallate significantly suppressed ^1^O_2_. Based on our results, it is concluded that ^1^O_2_ formation correlates with R^•^ formation on the donor side of PSII due to oxidation of organic molecules (lipids and proteins) by long-lived P680^•+^/TyrZ^•^. It is proposed here that the Russell mechanism for the recombination of two peroxyl radicals formed by the interaction of R^•^ with molecular oxygen is a plausible mechanism for ^1^O_2_ formation in the donor side photoinhibition of PSII.

## Introduction

Photosystem II (PSII) is a membrane pigment-protein complex located in the thylakoid membrane of oxygenic photosynthetic organisms (higher plant, algae and cyanobacteria). It is a homodimeric multisubunit complex, which is composed of proteins associated with various cofactors. Recent crystal structures of PSII from *Thermosynechococcus elongatus* and *Thermosynechococcus vulcanus* show that it is composed of 20 protein subunits, 35 chlorophylls, 12 carotenoids and 25 integral lipids per monomer [1]–[3]. It is involved in the conversion of light energy into chemical energy by water oxidation and plastoquinone reduction [4]–[7]. Light-driven water oxidation catalyzed by water-splitting manganese complex occurs via a step-wise release of four electrons and protons [8]–[12].

When higher plant, algae and cyanobacteria are exposed to high-light intensity illumination, PSII activity is inhibited in a process called photoinhibition [13]–[18]. Photo-inactivation of PSII is considered to be caused by damage to the D1 protein, one of the two proteins which formed a heterodimer with the D2 protein [19]–[22]. It is widely accepted that D1 damage is caused by two distinct mechanisms of photoinhibition i.e. the so called acceptor and donor side mechanism [19], [23]–[24]. In the acceptor side photoinhibition, over-reduction of the primary electron acceptor Q_A_ leads to its release from the binding site in the D2 protein [25]–[27]. In donor side photoinhibition, the formation of long-lived highly oxidizing molecules P680^•+^/TyrZ^•^ leads to the oxidation of the organic components such as proteins and lipids [18]–[19], [21].

It has been reported that different types of reactive oxygen species (ROS) are formed in both the acceptor and the donor side photoinhibition [28]–[30]. In the acceptor-side photoinhibition, singlet oxygen (^1^O_2_) is considered the main ROS responsible for PSII damage. The primary charge separation results in the formation of a primary radical pair ^1^[P680^•+^ Pheo^•−^] which leads to the formation of a secondary radical pair [P680^•+^Q_A_^•−^] by charge stabilization process. Under the complete or partial reduction of PQ pool, the reverse electron transport from Q_A_^•−^ to Pheo^•−^ forms ^1^[P680^•+^Pheo^•−^], which subsequently either recombines to the ground state P680 or converts to the triplet radical pair ^3^[P680^•+^ Pheo^•−^] by change in the spin orientation [18], [27], [30]–[31]. Singlet oxygen is generated by the interaction of molecular oxygen and triplet chlorophyll formed by the charge recombination of the triplet radical pair ^3^[P680^•+^ Pheo^•−^] [18], [28]–[30]. Singlet oxygen formation was shown by electron paramagnetic resonance (EPR) spin-trapping in the thylakoid membranes [32]–[33], PSII membranes [34], by chemical trapping [35] and phosphorescence at 1270 nm in PSII reaction center [36]. Apart from the radical pair recombination mechanism in the PSII reaction center, the formation of ^1^O_2_ occurs in the PSII antenna complex by intersystem crossing from the singlet excited chlorophyll via triplet-singlet energy transfer from the triplet chlorophyll [28]–[30]. It has been proposed that ^1^O_2_ can be generated from either weakly coupled or energetically uncoupled triplet chlorophylls in the PSII antenna complex [37]–[38]. Singlet oxygen formation in the isolated light harvesting complex II (LHCII) was shown by EPR spin trapping technique [39]–[40]. The authors concluded that ^1^O_2_ production in LHCII occurs as in the Type II photosensitization process. In this process, the triplet chlorophyll transfers its excitation energy to triplet molecular oxygen, while ^1^O_2_ is formed. In addition to ^1^O_2_ formation in the acceptor side photoinhibition, formation of the superoxide anion radical (O_2_^•−^) and the hydroxyl radical (HO^•^) has been demonstrated in PSII membranes [41]–[42].

In the donor side photoinhibition, the oxidation of proteins and lipids by highly oxidizing molecules P680^•+^/TyrZ^•^ results in the formation of carbon-centered radical (R^•^) [32]. Hydroxyl radical on PSII electron donor side was proposed to be formed by an unspecific reaction due to the photo-damage of thylakoid membrane by oxidizing reaction [32] and by the reduction of H_2_O_2_ formed on the PSII electron donor side [43]. In our best knowledge, there is no evidence on the formation of ^1^O_2_ on the donor side photoinhibition of PSII. It has been shown that the photoconsumption of molecular oxygen was increased six folds after the removal of water-splitting manganese complex from the PSII membranes [44]. Recently, lipid and protein hydroperoxides have been detected in Tris-treated PSII membranes [45]. It has been proposed that the loss of electron transport from water-splitting manganese complex to PSII reaction center leads to the oxidation of organic molecules by P680^•+^/TyrZ^•^ and consequently to the formation of organic R^•^ [44]–[47]. Apart from the above mention mechanism, manganese hypothesis has been recently assumed as another model in donor side photoinhibition [16], [48]. In this manganese hypothesis, the excitation of manganese by UV or visible light inhibits the electron transfer from water-splitting manganese complex to P680^•+^. Inactivation of water-splitting manganese complex occurs via the release of manganese and subsequently stabilizes the P680^•+^ for a longer period which leads to the photoinhibition of PSII.

In spite of the above mentioned *in vitro* mechanisms, a unifying model has been given to explain the photoinactivation of PSII under *in vivo* conditions [49]–[50]. It has been proposed that P680^•+^ has the capability for photoinactivation of PSII under steady state photosynthesis. The different ways of charge recombination have been regulated to the formation of primary radical pair ^1^[P680^•+^Pheo^•−^], which leads to the photoinactivation of PSII under steady state photosynthesis *in vivo*.

In this present study, the evidence for the formation of ^1^O_2_ in donor side photoinhibition is provided by using EPR spin-trapping spectroscopy. Light-induced ^1^O_2_ formation in Tris-treated PSII membranes was detected with hydrophilic spin trap compound TMPD. It is proposed here that the generation of ^1^O_2_ in the donor side photoinhibition of PSII occurs by the recombination of peroxyl radicals via the Russell mechanism.

## Results

### Singlet oxygen formation in Tris-treated PSII membranes

**Figure 1:**
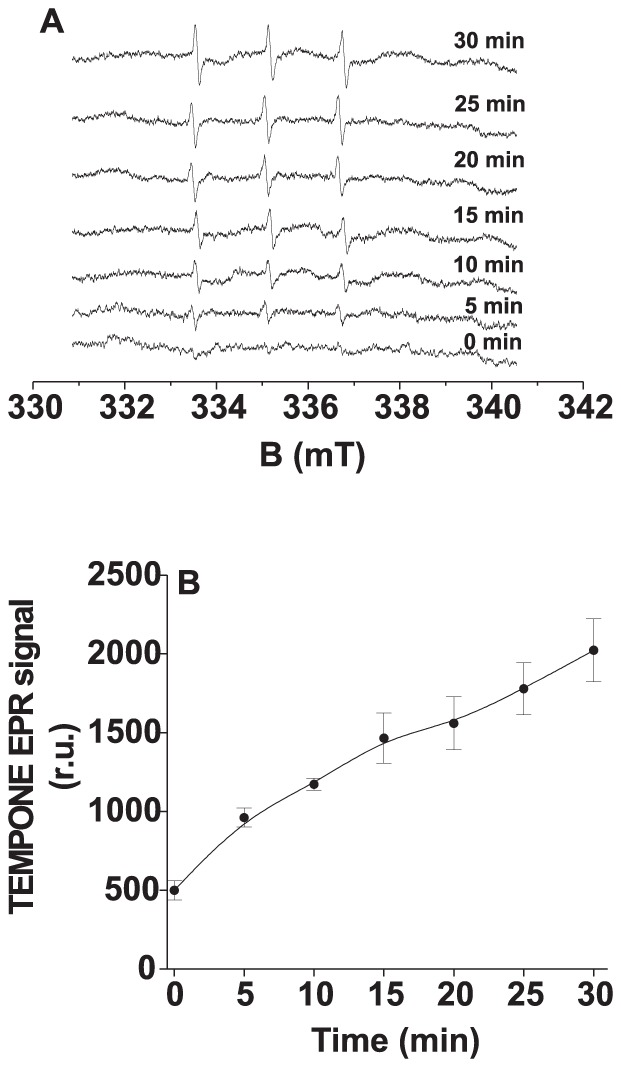
Light-induced TEMPONE EPR spectra measured in Tris-treated PSII membranes. [A] Tris-treated PSII membranes (200 µg Chl ml^−1^) were illuminated with white light (1000 µmol m^−2^ s^−1^) in the presence of 50 mM TMPD and 40 mM MES-NaOH (pH 6.5) for the time period as indicated in figure. [B] Time profile of TEMPONE EPR signal measured in Tris-treated PSII membranes under light illumination. The data represent the mean value (±SD) of at least three experiments.

In this study, light-induced formation of ^1^O_2_ in Tris-treated PSII membranes was measured by EPR spin-trapping technique. When spin-trapping was accomplished by utilizing the oxidation of lipophilic diamagnetic 2,2,6,6-tetramethylpiperidine (TEMP) by ^1^O_2_ which yields paramagnetic 2,2,6,6-tetramethylpiperidine-1-oxyl (TEMPO), no TEMPO EPR signal was detected (data not shown). The absence of TEMPO EPR signal was due to the oxidation of TEMPO by highly oxidizing species in Tris-treated PSII membranes. It is well known that under highly oxidizing conditions TEMPO is easily oxidized to oxoammonium salt [51]. To prevent the oxidation of paramagnetic TEMPO by highly oxidizing species formed on the PSII electron donor side, the spin-trapping was accomplished by utilizing the oxidation of hydrophilic diamagnetic 2, 2, 6, 6-tetramethyl-4-piperidone (TMPD) by ^1^O_2_ which yields paramagnetic 2, 2, 6, 6-tetramethyl-4-piperidone-1-oxyl (TEMPONE) (Fig. 1). Due to the fact that TMPD is a hydrophilic nitroxide spin trap, the nitroxyl radical TEMPONE monitors predominantly the formation of ^1^O_2_ in the polar phase. The addition of TMPD to Tris-treated PSII membranes in the dark resulted in the appearance of negligible TEMPONE EPR signal. The negligible TEMPONE EPR signal observed in non-illuminated Tris-treated PSII membranes was due to impurity of the spin trap. The exposure of Tris-treated PSII membranes to continuous white light resulted in the generation of TEMPONE EPR signal (Fig. 1A). Figure 1B shows that TEMPONE EPR signal increases gradually with illumination period. These observations indicate that illumination of Tris-treated PSII membranes results in ^1^O_2_ formation.

### Carbon-centered radical formation in Tris-treated PSII membranes

**Figure 2:**
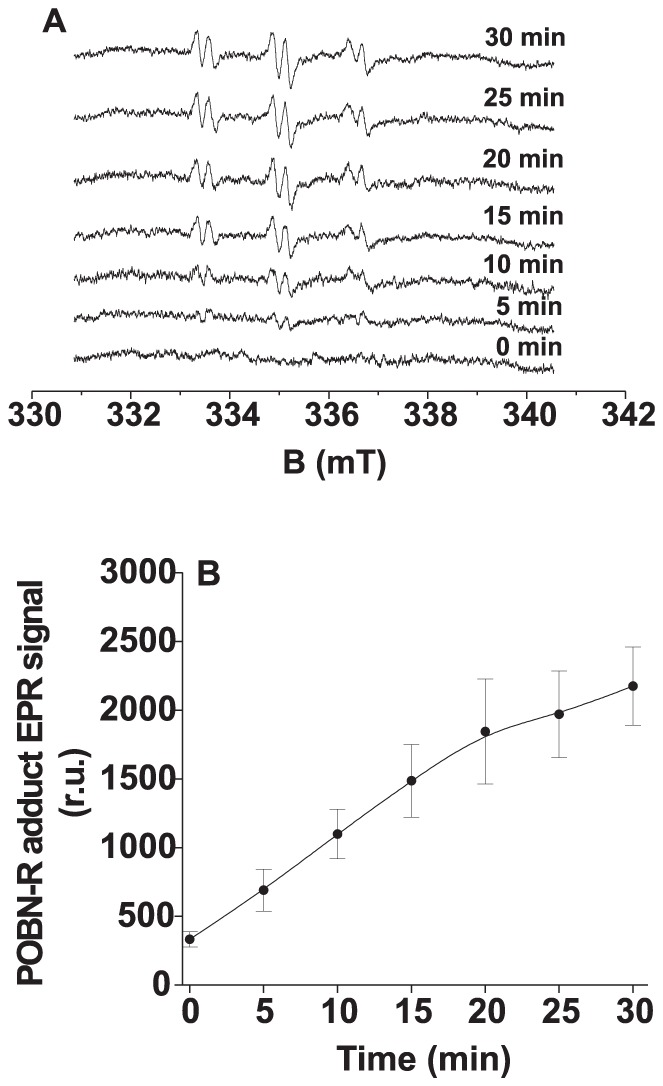
Light-induced POBN-R adduct EPR spectra measured in Tris-treated PSII membranes. [A] Tris-treated PSII membranes (200 µg Chl ml^−1^) were illuminated with white light (1000 µmol m^−2^ s^−1^) in the presence of 50 mM POBN and 40 mM MES-NaOH (pH 6.5) for the time period as indicated in figure. [B] Time profile of POBN-R adduct EPR signal measured in Tris-treated PSII membranes under light illumination. The data represent the mean value (±SD) of at least three experiments.

In order to detect the formation of R^•^ in Tris-treated PSII membranes, we used EPR spin-trapping technique using POBN (4-pyridyl-1-oxide-*N*-*tert*-butylnitrone) as the spin-trap compound. In the dark, no detectable POBN-R adduct EPR signal was observed in Tris-treated PSII membranes. The exposure of Tris-treated PSII membranes to continuous white light resulted in the generation of POBN-R adduct EPR signal (Fig. 2A). Figure 2B shows that the POBN-R adduct EPR signal increases with illumination of Tris-treated PSII membranes. These observations indicate that illumination of Tris-treated PSII membranes results in R^•^ formation.

### Effect of pH on ^1^O_2_ and R^•^ production in Tris-treated PSII membranes

**Figure 3:**
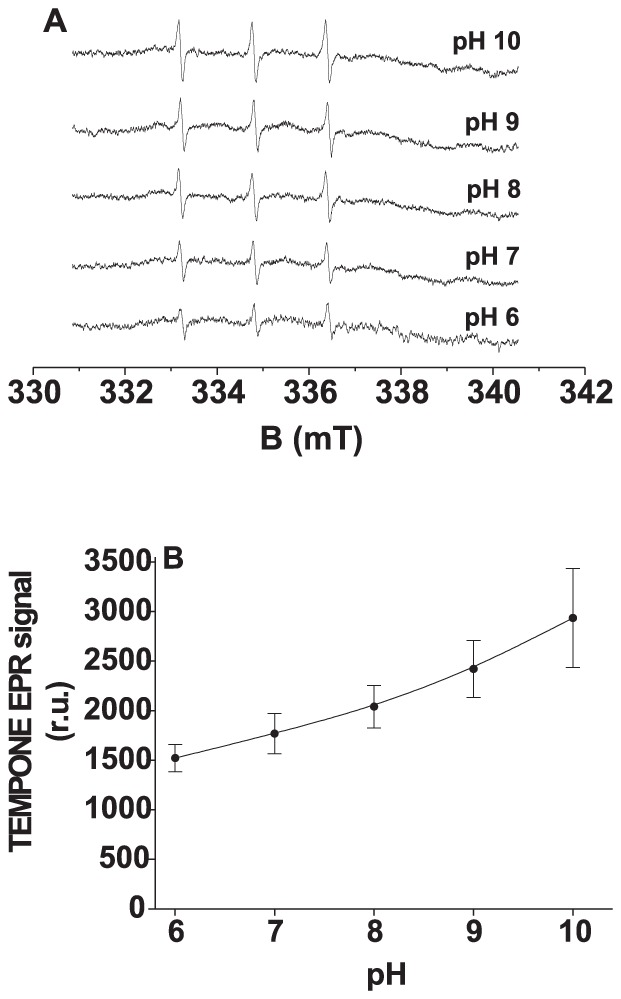
Effect of pH on TEMPONE EPR spectra measured in Tris-treated PSII membranes. [A] Tris-treated PSII membranes (200 µg Chl ml^−1^) were illuminated with white light (1000 µmol m^−2^ s^−1^) for 30 min at different pH as indicated in figure. The measurements were performed in 40 mM MES buffer (pH 6) or 40 mM HEPES buffer (pH 7) or 40 mM TRIS buffer (pH 8) or 40 mM CAPSO buffer (pH 9) or 40 mM CASP buffer (pH 10). [B] pH profile of TEMPONE EPR signal measured in Tris-treated PSII membranes under light illumination for 30 min. The intensity of EPR signal was evaluated as the relative height of central peak of the first derivative of the EPR absorption spectrum. The data represent the mean value (±SD) of at least three experiments.

**Figure 4:**
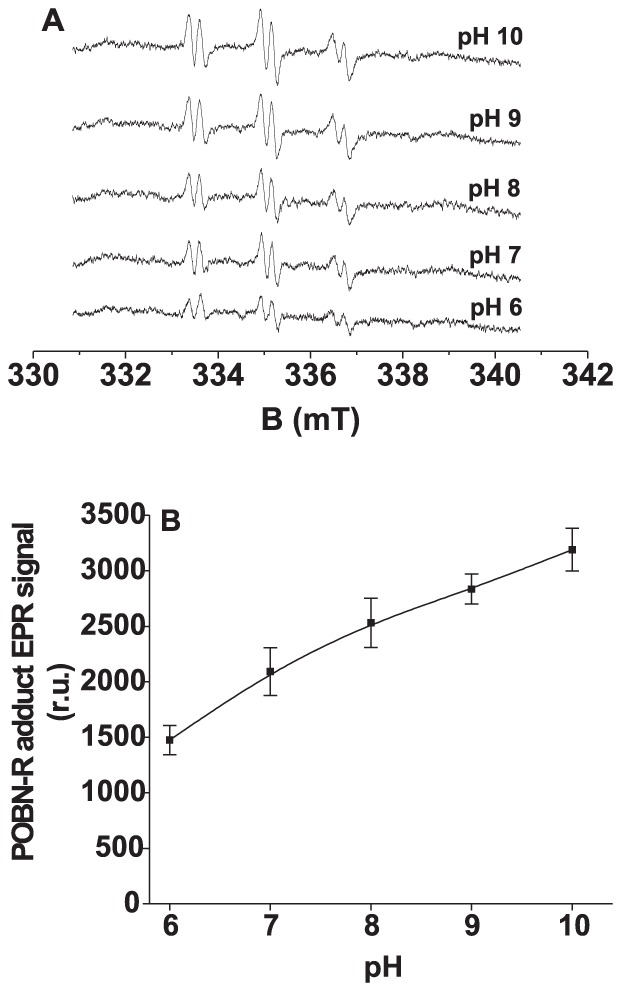
Effect of pH on POBN-R adduct EPR spectra measured in Tris-treated PSII membranes. [A] Tris-treated PSII membranes (200 µg Chl ml^−1^) were illuminated with white light (1000 µmol m^−2^ s^−1^) for 30 min at different pH as indicated in figure. The measurements were performed in 40 mM MES buffer (pH 6) or 40 mM HEPES buffer (pH 7) or 40 mM TRIS buffer (pH 8) or 40 mM CAPSO buffer (pH 9) or 40 mM CASP buffer (pH 10). [B] pH profile of POBN-R adduct EPR signal measured in Tris-treated PSII membranes under light illumination for 30 min. The intensity of EPR signal was evaluated as the relative height of central peak of the first derivative of the EPR absorption spectrum. The data represent the mean value (±SD) of at least three experiments.

To study the correlation between the light-induced ^1^O_2_ and R^•^ formation in donor side photoinhibition, the effect of pH on the formation of ^1^O_2_ and R^•^ was measured in Tris-treated PSII membranes. The pH increase caused a significant enhancement in TEMPONE (Fig. 3) and POBN-R adduct (Fig. 4) EPR signals. These observations indicate that the formation of ^1^O_2_ correlates with the formation of R^•^ in the donor side photoinhibition. The enhancement of TEMPONE and POBN-R adduct EPR signals at high pH reveals that the oxidation of organic molecules is involved in ^1^O_2_ formation in Tris-treated PSII membranes.

### Effect of propyl gallate on ^1^O_2_ and R^•^ production in Tris-treated PSII membranes

**Figure 5:**
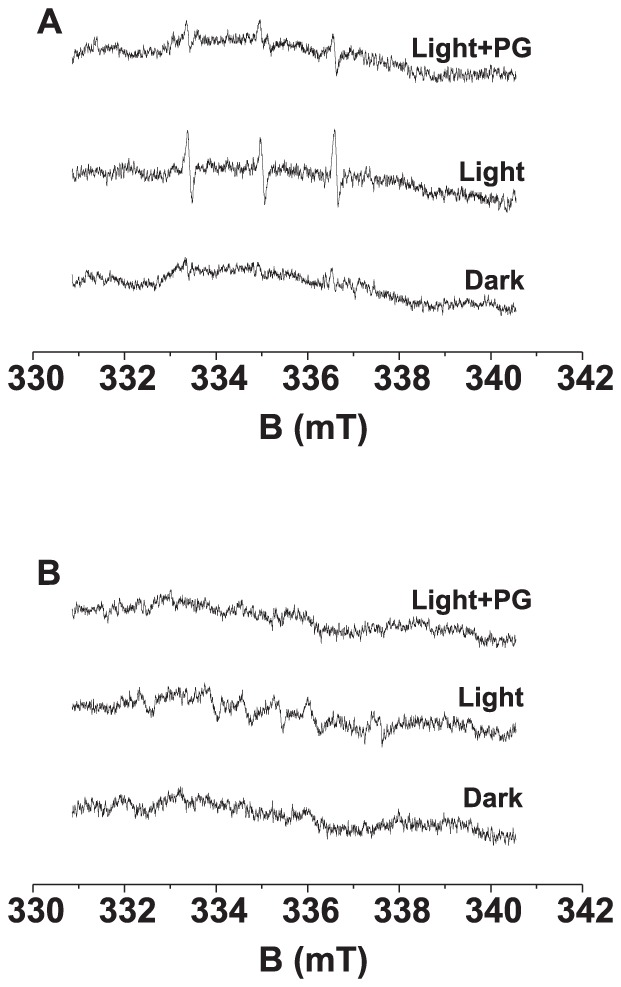
Effect of propyl gallate on TEMPONE and EMPO-R adduct EPR spectra measured in Tris-treated PSII membranes. [A] Tris-treated PSII membranes (200 µg Chl ml^−1^) were illuminated with white light (1000 µmol m^−2^ s^−1^) for 30 min in the absence (Light) and the presence (Light+PG) of 5 mM propyl gallate. Other experimental conditions were the same as described in Figure 1. [B] Tris-treated PSII membranes (200 µg Chl ml^−1^) were illuminated with white light (1000 µmol m^−2^ s^−1^) for 30 min in 40 mM MES-NaOH (pH 6.5), then added spin trap EMPO and measured the spectra without further illumination (dark). Due to the instability of EMPO-R adduct for a longer period illumination, Tris-treated PSII membranes were first illuminated 30 min in the absence of spin trap and then illuminated for additional 2 min in the presence of spin trap EMPO (Light). Tris-treated PSII membranes were first illuminated 30 min in the in the presence of 5 mM propyl gallate and then illuminated for additional 2 min by adding spin trap EMPO (Light+PG).

In order to confirm the correlation between light-induced ^1^O_2_ and R^•^ formation in the donor side photoinhibition, the effect of free radical scavenger propyl gallate on TEMPONE and EMPO-R adduct EPR signals was studied in Tris-treated PSII membranes. Figure 5A shows that the addition of propyl gallate to Tris-treated PSII membranes prior to illumination significantly suppressed the TEMPONE EPR signal. In order to study the formation of R^•^ in the presence of propyl gallate dissolved in ethanol, EMPO (5-(ethoxycorbonyl)-5-methyl-1-pyrroline N-oxide) spin trap was used instead of a POBN spin trap. As in the presence of ethanol, POBN reacts with α-hydroxyethyl radical (CH(CH_3_)HO^•^) formed by HO^•^ with ethanol, the detection of R^•^ by POBN in presence of ethanol is unfeasible. In the dark, no EMPO-R adduct EPR signal was observed in Tris-treated PSII membranes, whereas the exposure of Tris-treated PSII membranes to white light resulted in the generation of EMPO-R adduct EPR signal (Fig. 5B). The addition of propyl gallate to Tris-treated PSII membranes suppressed the EMPO-R adduct EPR signal in Tris-treated PSII membranes (Fig. 5B). These observations reveal that the scavenging of R^•^ by the addition of exogenous propyl gallate results in the suppression of ^1^O_2_ in Tris-treated PSII membranes.

## Discussion

It is well known that ^1^O_2_ is one of the most dangerous ROS in PSII, known to play a crucial role in the protein degradation of PSII under photoinhibitory conditions. The light-induced degradation of D1 protein occurs on both the electron acceptor and the electron donor side of PSII [19], [23], [52]. It is well established that ^1^O_2_ causes the damage of PSII in the acceptor site photoinhibition [18], [28], [30], [53], whereas the donor side photoinhibition occurs by highly oxidizing long lived molecules P680^•+^/TyrZ^•^ [18]–[19], [21], [23].

Using EPR spin-trapping spectroscopy, we demonstrated that the exposure of Tris-treated PSII membranes in the presence of hydrophilic spin trap TMPD to light illumination resulted in the formation of ^1^O_2_ (Fig. 1). However, the previous study showed that in the presence of lipophilic spin trap TEMP, ^1^O_2_ was not detected in Tris-treated thylakoid membrane [32], [54]. This may be due to instability of TEMPO in Tris-treated thylakoid membrane, caused by unspecific oxidizing species. Furthermore, we showed the formation of POBN-R adduct EPR signal in Tris-treated PSII membranes by light illumination (Fig. 2). This is in agreement with the previous proposal that R^•^ is formed on the donor side photoinhibition by the oxidation of organic molecules such as proteins and lipids [44].

It has been reported that the increase in photoconsumption of molecular oxygen at high pH is due to increased oxidation of organic molecules in Tris-treated PSII membranes [46]–[47]. In agreement with this, we showed here that the formation of ^1^O_2_ and R^•^ is enhanced in Tris-treated PSII membranes at high pH (Figs. 3 and 4). Similarly, we suggest that the increased oxidation of organic molecules at high pH enhances the formation of ^1^O_2_ via the Russell mechanism. Observations that the addition of propyl gallate to Tris-treated PSII membranes suppressed the formation of ^1^O_2_ (Fig. 5A) and R^•^ (Fig. 5B) reveals that the oxidation of organic molecules is involved in the production of ^1^O_2_ in Tris-treated PSII membranes.

**Figure 6:**
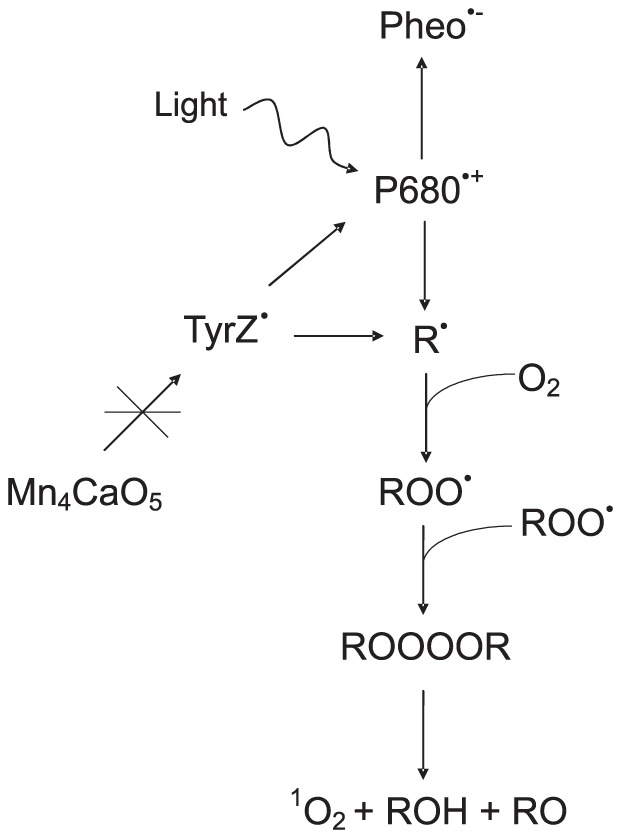
Proposed mechanism for the formation of ^1^O_2_ in the donor side photoinhibition of PSII. Carbon centered radical (R^•^) formed by oxidation of lipids and proteins react with molecular oxygen to form peroxyl radical (ROO^•^). Two peroxyl radicals react with the each other to form linear tetraoxide (ROOOOR) known to decompose to singlet oxygen (^1^O_2_), carbonyl (RO) and alcohols (ROH) via the Russell mechanism.

The formation of ^1^O_2_ via the Russell mechanism was reported in chemical systems [55]–[56]. Radical-mediated lipid and protein oxidation forms R^•^ known to form a peroxyl radical (ROO^•^) in the presence of molecular oxygen [57]–[58]. Singlet oxygen is produced via the decomposition of linear tetraoxide intermediate which is formed by the combination of two ROO^•^ [58]–[59]. Recently, it has been shown that ^1^O_2_ is formed by the enzymatic (cytochrome c and lactoperoxidase) decomposition of polyunsaturated lipid peroxide via the Russell mechanism [60]. Furthermore, it has been reported that the yield ^1^O_2_ is 10^3^–10^4^ times higher via decomposition of tetraoxide compared to the triplet excited carbonyl suggesting that the self reaction of peroxyl radical generates predominately ^1^O_2_ via Russell mechanism [61]–[62]. Similarly, we propose here that the light-induced oxidation of lipids and proteins by long lived highly oxidizing molecules P680^•+^/TyrZ^•^ results in the formation of R^•^. Carbon centered radical reacts with molecular oxygen to form a ROO^•^, which in turn oxidizes other organic molecules in the Tris-treated PSII membranes. The interaction of two ROO^•^ forms intermediate tetraoxide known to subsequently decompose into ^1^O_2_ [Fig. 6]. This pathway may lead to the formation of ^1^O_2_ as a byproduct via the Russell mechanism in the donor side photoinhibition. Apart from the Russell mechanism, the formation of ^1^O_2_ by Type II reaction i.e. excitation energy transfer from the triplet chlorophyll to molecular oxygen in the PSII antenna complex can not be completely excluded.

Crystal structure of PSII from *Thermosynechococcus elongatus* shows that PSII monomer is composed of 25 lipid molecules (11 monogalactosyldiacylglycerol (MGDG), 7 digalactosyldiacylglycerol (DGDG), 5 sulfoquinovosyldiacylglycerol and 2 phosphatidylglycerol) [2], [63]. The head group of lipid molecules faces towards the thylakoid membrane surface, whereas the tail is oriented to the interior of the membrane. Photosystem II reaction center is surrounded by polyunsaturated lipids that are mainly constituted by MGDG and DGDG. All the sulfoquinovosyldiacylglycerol and phosphatidylglycerol are located at the stromal side of the thylakoid membrane, whereas DGDG and MGDG are at the luminal side of the thylakoid membrane. Due to the more hydrophobic nature of MGDG and DGDG, lipid molecules are able to transfer across the membranes [3]. As the distance between the head of lipid molecule MGDG11 to the chlorophyll of P680 dimer (P_D1_) and the accessory chlorophyll (Chl_D1_) bound to D1 protein is around 5 Å [2], [64], MGDG11 could be the probable candidate for the initiation of lipid oxidation. Lipid molecules may provide structural flexibility around the PSII reaction center, thus facilitating the assembly and repair of PSII in the donor side photoinhibition [65]. Recently, it has been proposed that the lipid molecules provide an environment to keep molecular oxygen away from the PSII reaction center in order to prevent the oxidative damage of PSII [64].

In the PSII reaction center, chlorophyll dimer (P_D1_ and P_D2_) is distanced at 2.2 Å to the histidine residues D1-H198 and D2-H197, respectively [3]. The accessory chlorophylls Chl_D1_ and Chl_D2_ are hydrogen-bonded between the chlorine ring V and water molecule with a distance of 2.0 and 2.1 Å, respectively [3]. It has been reported that the midpoint redox potential of P680^•+^/P680 and TyrZ^•^/TyrZ redox couple ranges from 1.2 to 1.4 V and 1.1 to 1.2 V, respectively [5], [7], [10], [31], [66]–[67]. Due to the highest midpoint redox potential of P680^•+^/P680 redox couple, the chlorophyll dimmer could potentially be the main oxidizing species for the oxidation of organic molecules in the donor side photoinhibition. It is proposed here that the oxidation of lipids and proteins in the vicinity of highly oxidizing molecules P680^•+^/TyrZ^•^ leads to the formation ^1^O_2_ as a byproduct via the Russell mechanism.

## Materials and Methods

### PSII membranes preparation

PSII membranes were isolated from fresh spinach leaves purchased from a local market, using the method of Berthold et al. [68], with the modifications described in Ford and Evans [69]. PSII membranes suspended in a buffer solution containing 400 mM sucrose, 10 mM NaCl, 5 mM CaCl_2_, 5 mM MgCl_2_ and 50 mM MES-NaOH (pH 6.5) were stored at −80°C. Tris-treated PSII membranes were prepared by incubation of PSII membranes (1 mg Chl ml^−1^) in a buffer containing 0.8 M Tris–HCl (pH 8) for 30 min at 4°C, in the darkness with a continuous gentle stirring. After treatment, PSII membranes were washed twice in 400 mM sucrose, 10 mM NaCl, and 5 mM CaCl_2_ and 40 mM MES–NaOH (pH 6.5). Tris-treated PSII membranes suspended in the same buffer solution (pH 6.5) were stored at −80°C.

### EPR spin-trapping spectroscopy

EPR spin trapping is the direct and most sensitive technique for the detection of ROS in chemical and biological systems. As the life time of ROS is in the range from several ns to µs depending on the type of ROS, the direct detection of ROS by EPR spectroscopy is unfeasible. In EPR spin-trapping technique, unstable ROS interact with diamagnetic spin trap forming stable paramagnetic spin trap-radical adduct. As the life time of spin trap-radical adduct is in the range of several minutes to hours, the detection of EPR spin trap-radical adduct spectra is feasible by EPR spectroscopy.

Singlet oxygen was detected by hydrophilic spin trap compound TMPD (2, 2, 6, 6-Tetramethyl-4-piperidone) (Sigma) [70]. Oxidation of diamagnetic TMPD by ^1^O_2_, yields paramagnetic 2, 2, 6, 6-tetramethyl-4-piperidone-1-oxyl (TEMPONE) EPR signal. To eliminate impurity TMPD EPR signal TMPD was purified twice by vacuum distillation. Tris-treated PSII membranes (200 µg Chl ml^−1^) were illuminated in the presence of 50 mM TMPD and 40 mM MES-NaOH (pH 6.5) at 20°C. Illumination was performed with a continuous white light (1000 µmol photons m^−2^ s^−1^) using a halogen lamp with a light guide (Schott KL 1500, Schott AG, Mainz, Germany). After illumination, the sample was centrifuged at 5000×g for 3 min to separate TEMPONE from Tris-treated PSII membranes. It has been reported recently that the separation of two phases prevents the reduction of TEMPONE by a non-specific reducing component in thylakoid and PSII membranes [34], [71]. In this study, separation of the two phases was done to prevent the oxidation of TEMPONE by a non-specific oxidizing component in Tris-treated PSII membranes. After centrifugation, the upper phase was immediately transferred into the glass capillary tube (Blaubrand® intraMARK, Brand, Germany) and kept in liquid nitrogen until use. Prior to data collection, the capillary tube was taken away from the liquid nitrogen and EPR spin-trapping spectra were collected at room temperature.

Carbon-centered radicals were detected by either POBN (4-pyridyl-1-oxide-*N*-*tert*-butylnitrone) (Sigma) or EMPO (5-(ethoxycorbonyl)-5-methyl-1-pyrroline N-oxide) (Alexis Biochemicals, Lausen, Switzerland) [72]–[73]. In the POBN detection system, Tris-treated PSII membranes (200 µg Chl ml^−1^) were illuminated in the presence of 50 mM POBN and 40 mM MES-NaOH (pH 6.5). In the EMPO detection system, Tris-treated PSII membranes were first illuminated in the absence of spin trap for 30 min, whereas after illumination the sample was mixed with EMPO spin trap and further illuminated for 2 min. Illumination was performed with a continuous white light using a halogen lamp with a light guide (KL 1500 electronic, Schott, Germany). After illumination the sample was transferred into the glass capillary tube (Blaubrand® intraMARK, Brand, Germany) and EPR spectra were immediately recorded at room temperature. Spectra were recorded using EPR spectrometer MiniScope MS200 (Magnettech GmbH, Berlin, Germany). Signal intensity was evaluated as the height of the central peak of EPR spectrum. EPR conditions were as follows: microwave power, 10 mW; modulation amplitude, 1 G; modulation frequency, 100 kHz; sweep width, 100 G; scan rate, 1.62 G s^−1^.
